# Patient-derived xenograft models of gastrointestinal stromal tumors provide a ready-to-use platform for translational research

**DOI:** 10.1242/dmm.052225

**Published:** 2025-02-21

**Authors:** Luna De Sutter, Lore De Cock, Chao-Chi Wang, Daniël Gorgels, Karo Wyns, Kimberly Verbeeck, Ulla Vanleeuw, Thomas Douchy, Daphne Hompes, Joris Jaekers, Dirk Van Raemdonck, Isabelle Vanden Bempt, Maria Debiec-Rychter, Raf Sciot, Agnieszka Wozniak, Patrick Schöffski

**Affiliations:** ^1^ Laboratory of Experimental Oncology, Department of Oncology, KU Leuven, Leuven Cancer Institute, Leuven 3000, Belgium; ^2^ Department of Surgical Oncology, University Hospitals Leuven, Leuven Cancer Institute, Leuven 3000, Belgium; ^3^ Department of Abdominal Surgery, University Hospitals Leuven, Leuven 3000, Belgium; ^4^ Department of Thoracic Surgery, University Hospitals Leuven, Leuven 3000, Belgium; ^5^ Department of Human Genetics, KU Leuven, University Hospitals Leuven, Leuven 3000, Belgium; ^6^ Department of Pathology, University Hospitals Leuven, Leuven 3000, Belgium; ^7^ Department of General Medical Oncology, University Hospitals Leuven, Leuven Cancer Institute, Leuven 3000, Belgium

**Keywords:** Gastrointestinal stromal tumor, Patient-derived xenograft, Tumor model

## Abstract

Gastrointestinal stromal tumors (GISTs) are the most common mesenchymal malignancy of the gastrointestinal tract. Most GISTs harbor mutations in oncogenes, such as *KIT*, and are treated with tyrosine kinase inhibitors (TKIs), such as imatinib. Most tumors develop secondary mutations, inducing drug resistance against the available TKIs, requiring novel therapies. We established a GIST patient-derived xenograft (PDX) platform of GIST that can be used for preclinical drug testing. Tumor tissue from consenting GIST patients was transplanted subcutaneously to NMRI *nu/nu* mice. Once tumor growth was observed, the tumor was re-transplanted to a next generation of mice. Tumors were characterized histopathologically and molecularly at every re-transplantation and compared with the original patient tumor. We transplanted 112 tumor samples from 99 GIST patients, resulting in 12 established and well-characterized GIST models with different mutations and TKI sensitivity. Three models harbor secondary *KIT* mutations. One model is characterized by a primary, imatinib-resistant *PDGFRA* exon 18 p.D842V mutation. Our established platform of well-characterized GIST PDX models, covering the most relevant driver mutations, serves as an excellent tool for preclinical drug testing and tumor biology studies.

## Teaser

**Summary:** We established 12 gastrointestinal stromal tumor patient-derived xenograft models that are well characterized and reflect the clinic. They have already proven to be an excellent tool for preclinical drug testing.

## INTRODUCTION

Gastrointestinal stromal tumors (GISTs) are rare malignancies from mesenchymal origin that share molecular characteristics with the interstitial cells of Cajal, the pacemaker cells of the gastrointestinal tract (Blay et al., 2021). The annual incidence of GIST is 12 in 1 million in Europe (de Pinieux et al., 2021). Most GISTs are caused by gain-of-function mutations in the KIT proto-oncogene, receptor tyrosine kinase (*KIT*) or platelet-derived growth factor receptor alpha (*PDGFRA*) oncogenes. These mutations lead to self-activation of the receptor kinases for which these genes encode, resulting in deregulated proliferation and inhibition of apoptosis of the tumor cells (Blay et al., 2021). The most common molecular subtypes of GIST harbor *KIT* exon 11 mutations, occurring in ∼53-61% of all cases, or *PDGFRA* exon 18 mutations, found in 13-14% of patients (Wozniak et al., 2014; Blay et al., 2021). Only a fraction of GISTs have other underlying genetic abnormalities, such as mutations in neurofibromatosis type 1 (*NF1*) or B-Raf proto-oncogene, serine/threonine kinase (*BRAF*), or are characterized by succinate dehydrogenase (SDH) deficiency. The standard treatment for localized GIST is complete surgical excision of the lesion. Postoperative (adjuvant) therapy with the tyrosine kinase inhibitor (TKI) imatinib is used in patients at high risk of relapse after surgery, provided the tumor has an imatinib-sensitive genotype (Casali et al., 2022). Patients with inoperable, locally advanced and/or metastatic GIST are also treated with TKIs, and imatinib is the first-line treatment for GIST with *KIT* and *PDGFRA* mutations. A number of other drugs have regulatory approval for patients with GIST with disease progression or intolerance during treatment with imatinib. Avapritinib should be used for GIST with an underlying primary *PDGFRA* exon 18 p.D842V mutation, which is known to be insensitive to imatinib (Casali et al., 2022). Response rates to TKIs in the first line are very high; however, during treatment, the vast majority of patients will develop tumor progression. Progression can be caused by primary resistance to imatinib, in which GIST continues to progress despite the institution of therapy, or by acquired resistance to the TKI, mostly due to the acquisition of secondary mutations in *KIT* or *PDGFRA*, interfering with the inhibitory effect of imatinib (Antonescu et al., 2005; Wardelmann et al., 2006). Further lines of treatment in non-*PDGFRA* p.D842V GIST consist of sunitinib, regorafenib and ripretinib. The progression-free survival observed with these agents is relatively short, and disease control is achieved only for a limited period of time. Many patients with GIST who failed the commercially available TKI still have a good performance status and excellent organ function, and the high unmet medical need for novel therapies in these patients is illustrated by the common use of off-label therapies with other KIT or PDGFR inhibitors. Many patients get multiple lines of oral TKI treatments (Demetri et al., 2006, 2013; Blay et al., 2020; Casali et al., 2022).

Patient-derived xenograft (PDX) models are used for preclinical drug testing of new compounds *in vivo*. In typical PDX models, a tumor tissue fragment from a donor patient obtained during a surgical intervention or during a biopsy is transplanted to (partially) immunodeficient mice. If tumor growth is observed in these experimental animals, the growing lesions typically resemble the donor's tumors in terms of histopathological and genetic characteristics, and, moreover, they reflect a similar heterogeneity and 3D architectural structure as the original tissue. Additionally, PDX models show similar sensitivities to anti-cancer drugs as their donor patient's tumors in the clinical setting. PDX models are a well-established and reliable research tool with potential applications in preclinical drug testing and tumor biology studies (Fujii et al., 2020; Yoshida, 2020; Liu et al., 2023).

The Laboratory of Experimental Oncology (LEO), Department of Oncology, Leuven Cancer Institute, KU Leuven (Leuven, Belgium), a non-profit organization, has successfully established a comprehensive panel of soft tissue sarcoma PDX models, called the ‘XenoSarc’ platform (Cornillie et al., 2019). This platform consists of clinically common soft tissue sarcoma subtypes, such as GIST, leiomyosarcoma, dedifferentiated liposarcoma or myxofibrosarcoma, as well as rare subtypes, including pulmonary artery intimal sarcoma, myxoinflammatory fibroblastic sarcoma and PEComa, or subtypes with rare genetic aberrations, such as *CIC*-*DUX4* re-arranged round cell sarcoma. When the project started in 2004, the first aim was to establish preclinical models of GIST. Over the years, we continued establishing further GIST models, with the intention of creating a ready-to-use, comprehensive preclinical platform reflecting the clinical and genetic heterogeneity of this disease.

## RESULTS

### Patient and tumor sample characteristics

Since the launch of the XenoSarc platform in 2004, we consented 99 patients with GIST, who donated 112 GIST tumor samples to our laboratory for research purposes. The information of 14 samples from 13 patients had to be excluded from further analysis as the prebiopsy/presurgical working diagnosis was GIST, but the corrected, final diagnosis was leiomyoma (three patients), hemangioma (one patient), fibrous connective tissue of the liver (one patient) or normal gastric mucosa (one patient). In seven other cases, the final pathological diagnosis showed a different type of tumor, such as desmoid tumor (three patients), acinar cell carcinoma of the pancreas (two patients), adenocarcinoma (one patient) or hepatocellular carcinoma (one patient). From one tumor sample, no clinical information could be obtained. A total of 12 patients donated two tumor samples during the course of their disease, and one patient donated three samples. In the subsequent analysis, a total of 86 patients were included, donating 98 GIST samples. Nine tumor specimens were in an early stage of growth at time of the data cut-off and could not be evaluated as an established model. Therefore, for only 89 tumor samples from 78 patients, the following description and statistical correlation was made between clinical characteristics of the patient and donor tumor and establishment of the model.

Transplantation of 12 tumor samples (13.5%) led to an established PDX model, meaning that, after engraftment of the tumor sample (passage 3 and higher), stable histologic and molecular features for at least two passages were observed, similar to those of the original patient's tumor. The remaining 77 tumor samples did not grow in our mice and were considered unsuccessful.

Details and correlations between patient and tumor characteristics and establishment of a successful model can be found in Table 1. The median age of the donor patients at diagnosis of GIST was 59 years (range 9-87 years), and median age at sampling was 62 years (range 9-87 years). In 34 cases (38%), the patient had localized GIST at the time of tumor sampling (primary tumor in 29 cases and local relapse in five cases), and 55 donors (62%) had metastatic disease. Eleven out of 12 (92%) of our established models were derived from patients with metastatic disease at the time of sampling. A statistical correlation was found between the establishment of the model and disease status of the patient at sampling [localized GIST versus metastasized, generalized estimated equation (GEE), *P*=0.05], meaning that tumor samples from patients with metastasized GIST had a better establishment rate (Table 1). Most patients (*n*=48, 54%) had received systemic treatment prior to the biopsy or surgical intervention. In this pretreated cohort, all patients had received at least imatinib before sampling. Nine out of our 12 established models (75%) were derived from tumors of patients previously treated with systemic therapy, and eight of them were progressive at the time of sampling.

**Table 1.** Correlations between patient and tumor characteristics and establishment of a successful model

Characteristics	Number of cases/total number of samples with available information (%)	Number of established models (*n*=12)	*P*-value (GEE)
Sex			
Male	45/78 (58%)	8	*P*=0.28
Female	33/78 (42%)	4	
GIST disease status at time of tumor sampling			
Localized GIST (primary tumor or local recurrence)	34/89 (38%)	1	*P*=0.05
Metastatic GIST	55/89 (62%)	11	
Systemic treatment (at least imatinib) prior to tumor sampling			
Yes	48/89 (54%)	9	*P*=0.13
No	41/89 (45%)	3	
Procedure performed for obtaining tumor sample			
Biopsy	34/89 (38%)	5	*P*=0.79
Surgery	55/89 (62%)	7	
Origin of sample			
Primary tumor	31/89 (35%)	2	*P*=0.38
Local recurrence	10/89 (11%)	2	
Metastasis or ascites	48/89 (54%)	8	
Primary GIST location			
Gastrointestinal	61/71 (86%)	8	*P*=0.13
Extra-gastrointestinal*	10/71 (14%)	3	
Unknown		1	
Cellular morphology of patient's donor tumor			
Spindle cell	50/78 (64%)	6	*P*=0.78
Epithelioid	5/78 (6%)	1	
Mixed morphology	23/78 (30%)	4	
Unknown		1	
Mitotic count (per 50 high-power fields) in patient's donor tumor			
Low (≤5 mitosis/50 high-power fields)	32/67 (48%)	2	*P*=0.12
High (>5 mitosis/50 high-power fields)	35/67 (52%)	6	
Unknown		3	
KIT expression (IHC staining) in patient's donor tumor			
Positive	65/76 (85.5%)	9	*P*=0.71
Negative	11/76 (14.5%)	2	
Unknown		1	
Mutation in patient's donor tumor			
Primary *KIT*	48/81 (59%)	6	*P*=0.72
Primary and secondary *KIT*	20/81 (25%)	4	
*PDGFRA*	6/81 (7%)	1	
Other	7/81 (9%)	0	
Unknown		1	
Patient's GIST stage according to anatomy and AJCC staging at time of tumor sampling			
Stage I and II	20/89 (22.5%)	0	*P*<0.001
Stage III and IV	69/89 (77.5%)	12	
Development of GIST metastasis during the entire course of the patient's disease			
No metastasis	26/89 (29%)	0	*P*<0.001
Synchronous and metachronous metastasis^‡^			
Total with metastasis:	25/89 38/89 63/89 (71%)	12

GEE was used for statistical analysis, with *P*≤0.05 considered as significant. Information from models from tumor samples in the early stage (*n*=9), and missing or unknown information, were left out of this table and the statistical correlation analysis. *Extra-gastrointestinal includes the following locations: mesentery, peritoneum, prostate. ^‡^Synchronous metastasis is defined as metastasis at GIST diagnosis, and metachronous metastasis is defined as metastasis developed after completion of the initial curative treatment. AJCC, American Joint Committee on Cancer; GEE, generalized estimated equation; GIST, gastrointestinal stromal tumor; IHC, immunohistochemical; *KIT*, KIT proto-oncogene, receptor tyrosine kinase; *PDGFRA*, platelet-derived growth factor receptor alpha.

Routinely, we used solid tumor fragments obtained from surgery and biopsy specimens for implantation in our laboratory mice. Exceptionally, tumor cells were injected in mice: in one case, tumor cells were collected during ascites drainage; and in two cases, primary cells were isolated from tumor tissue obtained during a prior procedure (one biopsy and one surgery) and injected in mice at a later stage. These three cases did not lead to an established model. There was no difference in establishment rate between samples obtained during surgery (seven successful models out of 55 samples) and samples obtained from biopsies (five successful models out of 34 samples): 13% versus 15%, respectively. Tumor samples were retrieved from primary tumors (*n*=31; 35%), recurrent GIST (*n*=10; 11%), metastatic lesions [*n*=47; 53%; of which 22 were synchronous metastasis at diagnosis (47%)] and ascites fluid (*n*=1; 1%). Two out of our 12 established models (17%) originated from a primary tumor, two (17%) were from a local recurrence, and eight (67%) were from a metastatic lesion. The donors of the two models that were established from primary tumor samples both developed metastases. One had synchronous metastasis at the time of sampling, and the other had metachronous metastasis during the further course of the patient's disease. No correlation was found between the establishment of a model and the sample origin (primary tumor versus metastasis versus recurrence, GEE, *P*=0.38) (Table 1).

The primary location of GIST was mostly gastric (42%) and small intestinal (27%). Some extra-gastrointestinal primary GIST locations were seen, namely mesenteric, peritoneal and omental (each in three patients, 3%), and prostate in one patient. The primary location was unknown in seven patients (8%). Six tumor samples from gastric GISTs (16%) and two small intestinal GISTs (8%) led to successfully established models. Additionally, two mesenteric GISTs (67%) and one peritoneal GIST (33%) also led to an established model. No correlation was found between the establishment of a model and the primary location of the GIST (gastric and small intestinal versus extra-gastrointestinal locations, GEE, *P*=0.13) (Table 1).

**Fig. 1.**
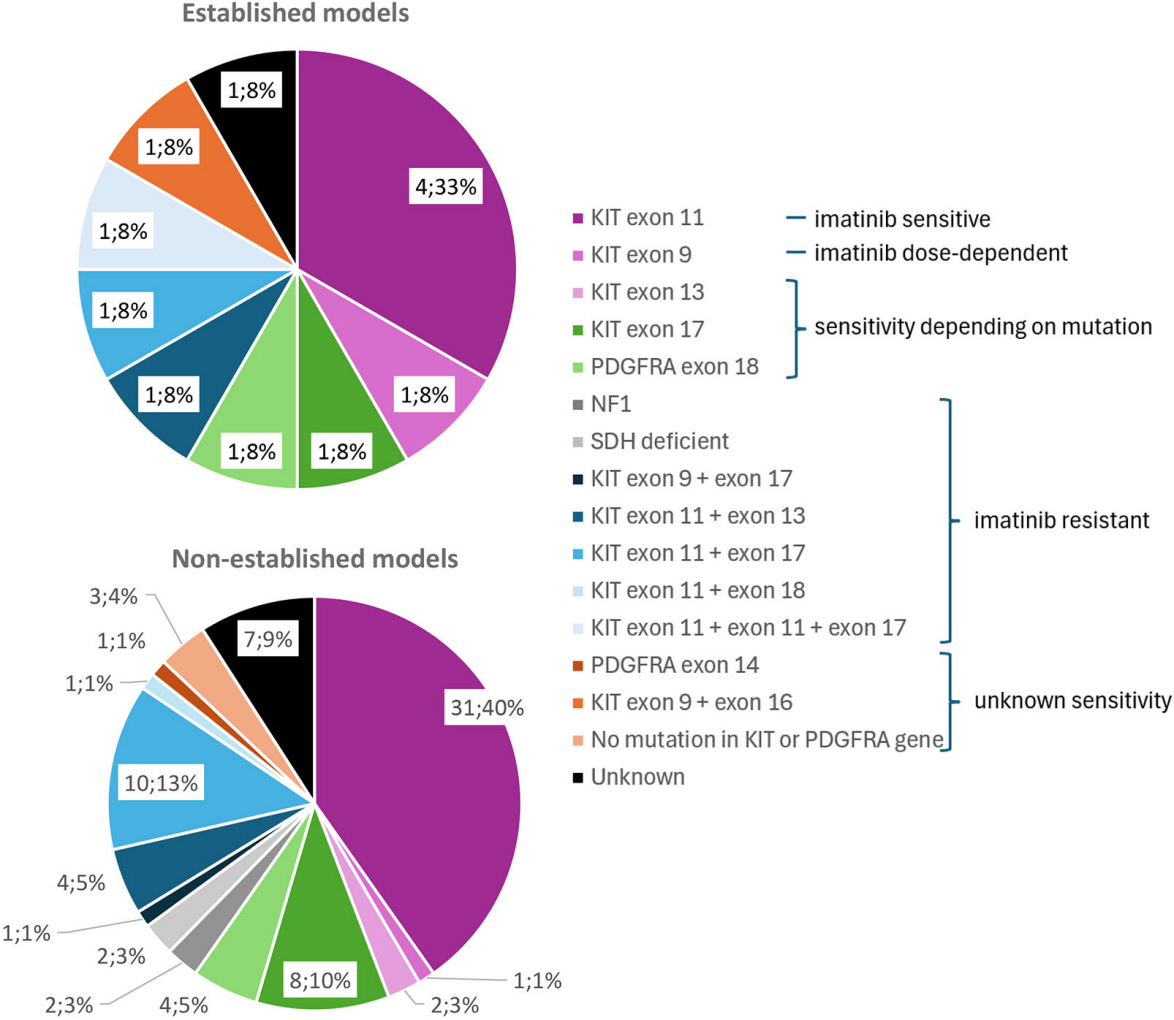
**Mutational analysis results from patient donor tumors according to establishment of patient-derived xenograft (PDX) model.** Pie chart showing the driver mutations found in the original patient cohort of gastrointestinal stromal tumor (GIST) samples that were established as PDX models (top), compared to a pie chart of the samples for which we were not able to establish a PDX model (bottom). Mutations are classified by imatinib sensitivity as known from the literature. The charts show the number and percentage of transplanted samples per driver mutation. Numbers are small; therefore, statistical conclusion cannot be drawn. It does seem, however, that driver mutations do not have a major influence on establishment rates. *KIT*, KIT proto-oncogene, receptor tyrosine kinase; *NF1*, neurofibromatosis type 1; *PDGFRA*, platelet-derived growth factor receptor alpha; SDH, succinate dehydrogenase.

Histological characterization of the tumor samples revealed a spindle cell morphology in 50 cases (56%), epithelioid morphology in five tumors (6%) and a mixed appearance in 23 cases (26%). For 11 tumor samples (12%), this information was missing. No statistical correlation could be found between the establishment of a model and the histological subtype of GIST (GEE, *P*=0.78). Thirty-two tumor samples (48%) showed a low mitotic rate [grade 1 (G1); ≤5 mitoses/50 high-power fields] compared to 35 tumor samples (52%) with high mitotic rate [grade 2 (G2); >5 mitoses/50 high-power fields]. More models were established from tumors with a higher mitotic rate (50%) than from those with a low mitotic rate (17%), but this trend was not statistically significant. In 22 tumor samples (22%), the mitotic rate was unable to be assessed or this information was missing, including in three tumor samples of established models. Most original tumor samples showed KIT positivity in immunohistochemical (IHC) analysis (65 tumor samples; 85.5%). For 13 tumor samples, this information was missing (13%). Discovered on GIST 1 (DOG-1; also known as ANO1) showed IHC positivity in all tumor samples for which information was available. Cluster of differentiation 34 (CD34) showed IHC positivity in 27 out of 45 tumor samples (60%) for which this information was available. Mutational analysis results from the tumor samples can be found in Fig. 1 and Table 2. The most common mutation present in the donors’ tumor samples was *KIT* exon 11. There were more tumor samples carrying only a primary *KIT* mutation, the driving force for GIST tumorigenesis, than tumor samples carrying secondary mutations, mutations appearing after treatment. In the 36 *KIT* mutated GIST samples from patients that were systemically treated before sampling, 18 samples (50%) showed primary and secondary mutations. More models were established from donor tumor samples carrying a *KIT* mutation than from those carrying a *PDGFRA* mutation, corresponding with the lower incidence and less aggressive behavior of *PDGFRA*-related disease. Of the non-*KIT* non-*PDGFRA* mutated tumor samples, no models could be established. Among the 12 established models, only three tumor samples (25%) had not been treated with systemic therapy prior to sampling. The nine other established models (75%) were treated before sampling; three of these tumor samples carried only primary *KIT* mutations and four had primary and secondary mutations. One was a *PDGFRA*-mutated GIST, and from one model the clinical information about mutational status of the tumor sample was missing. These nine tumors were progressive under TKI treatment at tumor sampling. No correlation was found between the establishment of a model and the mutational subtype (GEE, *P*=0.72) (Table 1).

**Table 2.** Mutational analysis results from patients' donor tumors

Mutation(s)	Number of samples	Number of established models
*KIT*	68 (76%)	10
Primary	48	6
*KIT* exon 9	9	1
*KIT* exon 11	35	4
*KIT* exon 13	2	1
*KIT* exon 17	2	0
Primary+secondary	20	4
*KIT* exon 9+exon 16	1	1
*KIT* exon 9+exon 17	1	0
*KIT* exon 11+exon 11+exon 17	1	1
*KIT* exon 11+exon 13	5	1
*KIT* exon 11+exon 17	11	1
*KIT* exon 11+exon 18	1	0
*PDGFRA*	6 (7%)	1
*PDGFRA* exon 14	1	0
*PDGFRA* exon 18	5	1
Other	7 (8%)	0
*NF1*	2	0
SDH deficient	2	0
No mutation in *KIT* or *PDGFRA* gene, no other genes tested	3	0
Unknown	8 (9%)	1

In the hospital, patients' donor tumors were evaluated for mutational analysis at the time of biopsy or surgical resection (Vanden Bempt et al., 2020). We received samples of these tumors for xenografting. A total of 85 patients donated a total of 98 GIST tumor samples. The most common mutation present in the donor tumor samples was *KIT* exon 11. No information on mutational analysis was available in the clinical file for eight tumor samples. *KIT*, KIT proto-oncogene, receptor tyrosine kinase; *NF1*, neurofibromatosis type 1; *PDGFRA*, platelet-derived growth factor receptor alpha; SDH, succinate dehydrogenase.

**Fig. 2.**
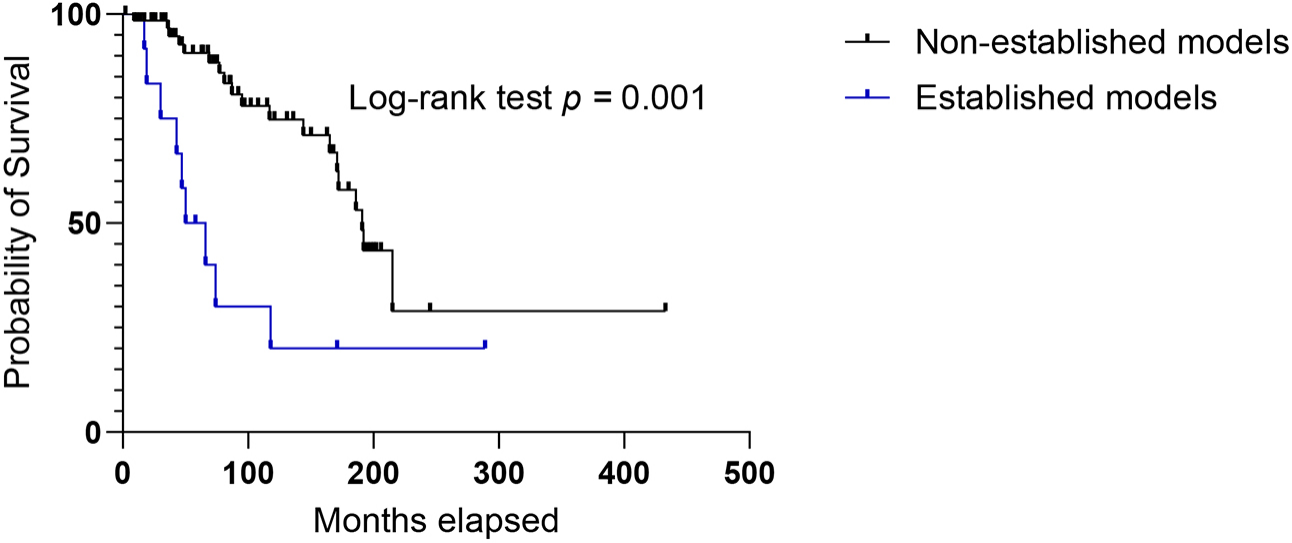
**Kaplan–Meier analysis of overall survival of the donor patients by establishment status of the PDX model.** Statistical significance was calculated using log-rank test, with *P*≤0.05 considered as significant. Overall survival was defined as the time between first GIST diagnosis and death from the disease. For this survival analysis, only the establishment of the chronologically first-received sample from patients that donated more than one sample was taken into account. Death from an unrelated cause was considered to be a censored observation in our study design. Early-stage models were not included in the correlation or survival analysis.

At the moment of tumor sampling, most patients presented with stage IV disease (58 cases; 65%). Of their tumor samples, 11 (19%) led to established models, which is the majority of our established models (92%). The other established model originated from a tumor sample of a GIST stage IIIB. A correlation was found between the establishment of a model and the disease stage at sampling (stage III+IV versus stage I+II, GEE, *P*<0.001), meaning that tumor samples from patients with advanced GIST had a better establishment rate than did those from patients with earlier-stage GIST (Table 1). No tumor samples from patients with stage I and stage II disease led to establishment of models, and none of the T1 (tumor ≤2 cm) or T2 (tumor >2 cm but ≤5 cm) GISTs led to an established model. The tumors of 41 patients progressed after initial treatment. At first progression, one patient developed progression of a residual lesion after surgery, 15 patients had local recurrence, which is defined here as recurrent GIST at the site of the initial surgical resection site, and 25 patients developed metastasis. A total of 63 patients in our cohort (71%) developed metastasis during the course of their disease: 25 patients (28%) had synchronous metastasis at diagnosis, and 38 patients (43%) developed metachronous metastasis after completion of the initial curative treatment. All donors of our established models initially presented with or eventually developed GIST metastasis (100%). A statistical correlation was found between the establishment of a model and the development of metastasis during the entire course of the patient's disease (synchronous and metachronous metastasis versus no metastasis, GEE, *P*<0.001) (Table 1). Patients whose tumor led to a successfully established model had worse overall survival than patients whose tumor did not grow in our laboratory mice (*P*=0.001, log rank test) (Fig. 2). The median overall survival of patients with successfully established models since first GIST diagnosis was 58 months, compared with 191 months for patients whose model was not established.

### Established GIST PDX models

**Fig. 3.**
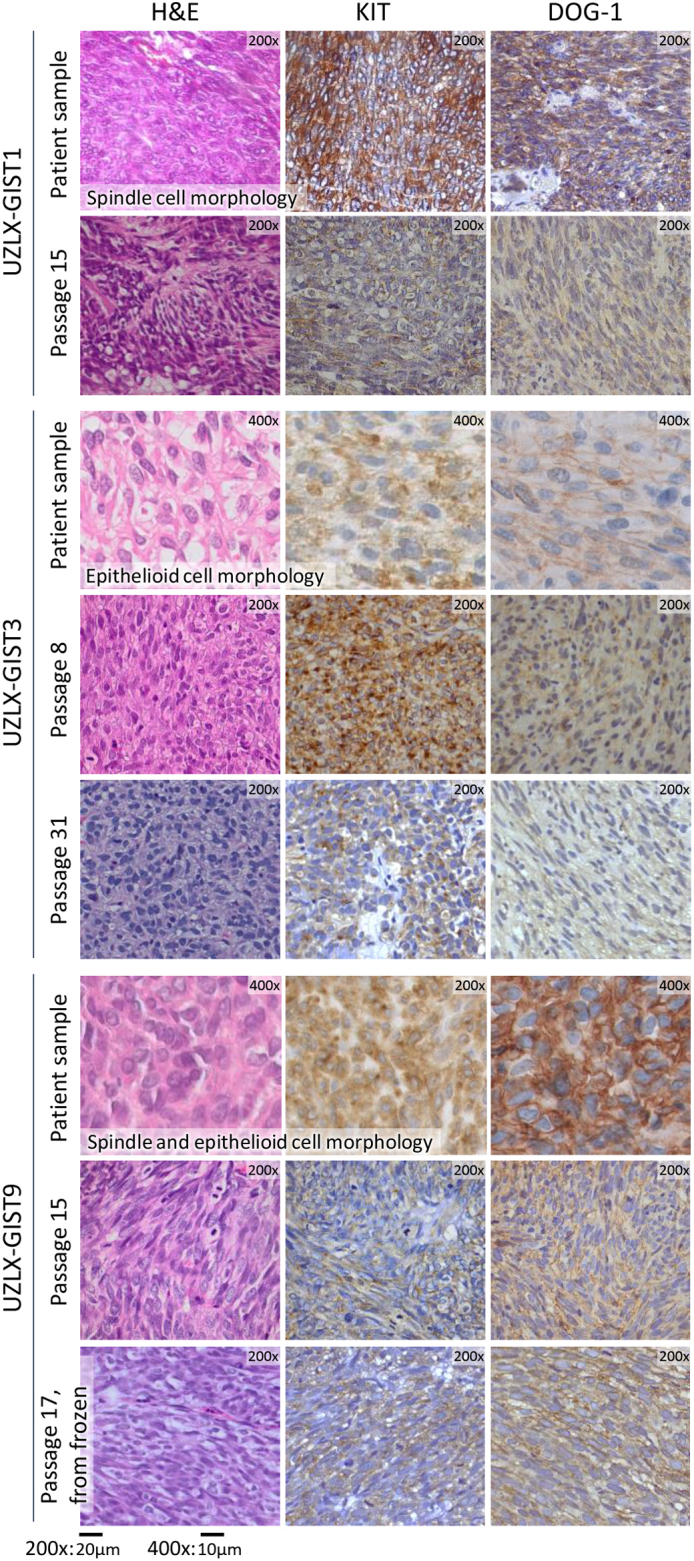
**Histopathological characteristics of three of the established GIST PDX models.** H&E and immunohistochemical staining examples of the donor tumors and the corresponding GIST PDX models. The characteristics of the models are identical to those of their donor tumor and remain stable over passaging. DOG-1, discovered on GIST 1; H&E, Hematoxylin and Eosin; *KIT*, KIT proto-oncogene, receptor tyrosine kinase.

An overview of the clinical characteristics of the donors and their tumors per established GIST PDX model can be found in Table S1. For all established models, human origin was validated, confirmed on each passage by the presence of HLA class I histocompatibility antigen, A alpha chain (HLA-A) in IHC analysis. Seven models (58%) showed spindle cell morphology, one model (8%) showed epithelioid morphology, and four models (33%) showed mixed morphology. These characteristics are identical to the donor tumor's morphology, except for one (UZLX-GIST25), for which this information on the donor's tumor sample is missing. Eleven models (92%) were strongly KIT positive, and all models were DOG-1 positive, in IHC analysis. In one model (UZLX-GIST73), the donor tumor analyzed at the hospital's pathology department was KIT negative but had been weakly positive in our model since passage 0. Throughout passaging in the mice, the IHC characteristics were preserved in all models. Histopathological characteristics per established model can be found in Table S2, and some examples of histopathological images are shown in Fig. 3.

In our platform, out of the 12 established models, there are eight models (67%) carrying only a primary mutation, including one (UZLX-GIST70) with a *PDGFRA* exon 18 p.D842V mutation. Four models (33%) carry secondary mutations, emerging during or after treatment, of which one model (UZLX-GIST9) has a triple *KIT* mutation, one primary mutation in *KIT* exon 11 and two secondary mutations – in *KIT* exon 11 (nonsense) and in *KIT* exon 17. The mutational characteristics, clinical and *in vivo* response to TKIs, and the *in vivo* growth rate of the 12 established GIST PDX models, as well as the clinical responses of the donor patients to TKIs, can be found in Table 3. Eleven models (92%) show the same mutational characteristics as their donor tumor, and these characteristics remain stable over passaging. In one case (UZLX-GIST76), we noticed different mutations when comparing the donor tumor pathology report and our analysis of the received and transplanted tumor parts. Clinical mutational analysis through targeted next-generation sequencing of the patient's sample showed a *KIT* exon 11 and exon 13 mutation; in the received and subsequently passaged samples, a *KIT* exon 11 and exon 17 mutation was confirmed through bi-directional Sanger sequencing. At sampling, the patient showed progressive disease, 1.5 years after imatinib treatment, with initially partial response.

**Table 3.** Established GIST PDX model characteristics

PDX model	Primary mutation Secondary mutation(s)	Clinical tumor treated systemically before sampling	Clinical response to TKIs	*In vivo* TKI sensitivity	*In vivo* tumor growth rate*
UZLX-GIST1	*KIT*: p.V560D (exon 11)	No	Imatinib resistance after tumor sampling	Imatinib/sunitinib sensitive (Van Looy et al., 2015)	4 months
UZLX-GIST2B	*KIT*: p.A502_Y503dup (exon 9)	Yes	Imatinib resistance before tumor sampling	Imatinib dose-dependent sensitivity; sunitinib sensitive (Gebreyohannes et al., 2019b)	3 months
UZLX-GIST3	*KIT*: p.W557_V559delinsF (exon 11)	No	Imatinib/sunitinib/regorafenib resistance after tumor sampling	Imatinib/sunitinib sensitive (Gebreyohannes et al., 2019b)	2 months
UZLX-GIST4	*KIT*: p.K558_G565delinsR (exon 11)	No	Imatinib/sunitinib resistance after tumor sampling	Imatinib/sunitinib sensitive (Gebreyohannes et al., 2016)	2 months
UZLX-GIST9	*KIT*: p.P577del (exon 11) *KIT*: p.W557LfsX5 (exon 11) *KIT*: p.D820G (exon 17)	Yes	Imatinib/sunitinib/regorafenib resistance before tumor sampling	Imatinib/sunitinib resistant; regorafenib sensitive (Van Looy et al., 2014)	2 months
UZLX-GIST25	*KIT*: p.K642E (exon 13)	Yes	Imatinib/sunitinib/regorafenib resistance before tumor sampling	Imatinib dose-dependent sensitivity; sunitinib sensitive (De Sutter et al., 2023)	2 months
UZLX-GIST41	*KIT*: p.L576P (exon 11)	Yes	Imatinib/sunitinib resistance before tumor sampling; regorafenib/pazopanib resistance after tumor sampling	Not tested yet	4-5 months
UZLX-GIST47	*KIT*: p.V560D (exon 11) *KIT*: p.Asp820Gly (exon 17)	Yes	Imatinib/trabectedin resistance before tumor sampling; avapritinib/sunitinib/regorafenib/ripretinib resistance after tumor sampling	Not tested yet	5-6 months
UZLX-GIST70	*PDGFRA*: p.D842V (exon 18)	Yes	Imatinib resistance, avapritinib sensitive before tumor sampling	Not tested yet	5-6 months
UZLX-GIST73	*KIT*: p.Ala502_Tyr503dup (exon 9) *KIT*: p.Leu783Val (exon 16)	Yes	Imatinib/sunitinib/regorafenib resistance before tumor sampling; ripretinib resistance after tumor sampling	Not tested yet	3-4 months
UZLX-GIST76	*KIT*: p.Trp557_Lys558del (exon 11) *KIT*: p.Tyr823Asp (exon 17)	Yes	Imatinib sensitive before and after tumor sampling	Not tested yet	2 months
UZLX-GIST77	*KIT*: K558_E562del (exon 11)	Yes	Imatinib sensitive before and after tumor sampling	Not tested yet	3 months

*The *in vivo* tumor growth rate is defined as the duration from transplantation of the tumor to reaching a medium- to large-sized tumor (∼500 mm³) that is ready for re-transplantation during consecutive passaging. GIST, gastrointestinal stromal tumor; *KIT*, KIT proto-oncogene, receptor tyrosine kinase; PDGFRA, platelet-derived growth factor receptor alpha; PDX, patient-derived xenograft; TKI, tyrosine kinase inhibitor.

In half of our models, TKI sensitivity has so far been tested in drug sensitivity studies *in vivo* (Van Looy et al., 2014, 2015; Gebreyohannes et al., 2016; Gebreyohannes et al., 2019b; De Sutter et al., 2023) (Table 3), showing imatinib resistance in UZLX-GIST9, leading to tumor growth under imatinib treatment and imatinib dose-dependent sensitivity in UZLX-GIST2B and UZLX-GIST25, leading to tumor volume stabilization under normal dose imatinib treatment compared to that in untreated tumors. These three models were established from tumor samples from patients pretreated with TKIs, and we saw similar response behavior *in vivo* compared to the clinical TKI sensitivity prior to sampling. The other tested models (UZLX-GIST1, UZLX-GIST3 and UZLX-GIST4) showed imatinib sensitivity, leading to tumor shrinkage under imatinib treatment. These three models were established from tumor samples from treatment-naïve patients. In these donor patients, we saw tumor progression under at least imatinib treatment, after sampling, later on in the course of their disease. Lastly, in general, from ten models, the patient's tumor showed clinical imatinib resistance during the course of their disease, including the period before and after sampling and establishment of the model.

The *in vivo* tumor growth rate is defined as the duration from transplantation of the tumor to reaching a medium- to large-sized tumor (∼500 mm³) that is ready for re-transplantation during consecutive passaging. This growth rate in our models varies between 2 and 6 months, depending on the model. At first passages, these growth rates tend to be longer than in later passages. Table 3 shows the average growth rate at later passages.

To study the genomic stability between passages per model, copy-number analysis was performed using low-coverage whole-genome sequencing. Results are available from six of the GIST PDX models. For all studied models, we observed stable copy-number profiles throughout passaging.

Additionally, for all established models, we also performed RNA sequencing. Individual RNA-sequencing data per model are available, including the most highly expressed genes per model and comparisons with our other soft tissue sarcoma PDX models.

These models have already proven to be clinically relevant. They have been used in multiple preclinical drug testing studies (Table S3), which have already provided a preclinical rationale and supportive evidence for a number of clinical trials. Partially based on our *in vivo* work, avapritinib is now approved in the clinic for the treatment of advanced GIST with the specific *PDGFRA* p.D842V mutation (Gebreyohannes et al., 2019b). Similarly, IDRX-42 is currently being tested in a phase I trial (NCT05489237) (De Sutter et al., 2023).

### Tissue microarrays (TMAs)

We developed TMAs from our GIST PDX models, using *ex*-mouse tumor samples from different passages. At the time of writing, ten TMAs are available, with two to three cores per tumor sample included in the TMAs. The TMAs were stained with Hematoxylin and Eosin (H&E) and for GIST-related markers (KIT, DOG-1, CD34), in order to evaluate the presence of GIST cells in the cores. Some of the TMAs have already been used in biomarker studies and were found to be useful for target identification in preparation of some *in vivo* work. The TMA collection is available for collaborative research projects.

## DISCUSSION

Since 2004, our laboratory has been collecting tumor samples from patients with soft tissue sarcoma, starting with GIST samples for xenografting into NMRI *nu/nu* mice to create PDX models. Over the past 20 years, we were able to collect 98 confirmed GIST samples from 85 patients, resulting in 12 established GIST PDX models and nine models in early stage, with a variety of mutations and a heterogeneous TKI sensitivity profile. In this paper, we present patient and tumor characteristics of all received GIST samples and correlations between these characteristics and the establishment of a PDX model. Patient characteristics of all the donors resemble those of the general GIST population. There was a slightly higher incidence of GIST in males in our cohort, and the median age at diagnosis of our population was 59 years (Casali et al., 2022). We found a similar incidence of primary GIST anatomical locations as that reported in the literature (Miettinen and Lasota, 2006; Joensuu et al., 2012; Wozniak et al., 2014; Blay et al., 2021). We have, however, not received any tumor samples from primary GIST locations such as the colon, rectum or esophagus, which usually account for ∼5-6% of primary locations (Miettinen and Lasota, 2006; Joensuu et al., 2012; Blay et al., 2021). Interestingly, one GIST sample from a primary and untreated tumor originated from the prostate, which has been described in the literature (Reinke et al., 2016; Lu et al., 2021; Yadav et al., 2021) but is very rare. Unfortunately, this tumor sample did not lead to an established model. Remarkably, from the three mesenteric tumor samples and three peritoneal tumor samples, we were able to establish two models and one model, respectively, thus accounting for high establishment rates. Although these numbers are still too small to draw any firm conclusions, mesenteric, peritoneal and omental GISTs all belong to the category of GISTs of extra-gastrointestinal locations, and they are known to have a worse overall survival than that of GISTs of gastrointestinal location. It has already been discussed in the literature, mostly for epithelial cancers, that generally higher-grade, more aggressive tumors engraft more easily than lower-grade, less aggressive ones (Byrne et al., 2017), and this study confirmed this in GISTs. We indeed observed that tumor samples from patients with more advanced GIST [metastatic disease, American Joint Committee on Cancer (AJCC) disease stage III to IV disease], as well as tumor samples from patients who developed metastasis during the course of their disease, have a statistically higher chance of becoming an established model in the laboratory mice than do tumor samples from patients with earlier-stage GIST. Additionally, patients whose tumor samples led to an established model had a statistically significantly worse overall survival than patients whose tumor did not grow in our laboratory mice. These survival rates can, of course, be biased by the fact that these patients had higher disease stage and had metastasis at some point during their disease, but these results also show that the clinically more aggressive tumors engraft better in laboratory mice than do less aggressive ones. These are also the tumors that frequently lack sufficient treatment options for patients, and thus these PDX models remain valuable for testing novel drugs and combinations. In the future, it could also be interesting to try to improve the establishment rate for less aggressive GIST.

During the early days of the project, the laboratory focused on xenografting GIST samples from imatinib-resistant tumors to facilitate the *in vivo* testing of next-generation TKIs. Altogether, in our cohort of all collected GIST tumor samples, we saw a similar distribution of mutated genes (*KIT*, *PDGFRA* and others) as that reported in the literature (Joensuu et al., 2012; Klug et al., 2022), but, for secondary resistance mutations, we observed a higher number of *KIT* exon 17 (13 samples) mutations and *KIT* exon 13 (five samples) mutations, compared to the incidences of these molecular subtypes reported in the literature (Serrano et al., 2019). Some rare secondary mutations were found in the tumors of our cohort, such as secondary mutations in *KIT* exon 11, which is predicted to be a gain-of-function mutation, and exon 16 and exon 18, which are known to be associated with imatinib and sunitinib resistance (Vetto, 2009; Corless et al., 2011; Díaz Delgado et al., 2011). One PDX model could be established from a tumor sample with a secondary *KIT* exon 16 mutation and a primary *KIT* exon 9 mutation (UZLX-GIST73), and an imatinib-resistant PDX model could be established with a primary *KIT* exon 11 mutation, and secondary *KIT* exon 11 and exon 17 mutations (UZLX-GIST9). The second model has already been used in a number of preclinical studies testing novel compounds, confirming its TKI resistance (Van Looy et al., 2014; Gebreyohannes et al., 2016, 2019a,b; Pulkka et al., 2019; Wang et al., 2020; Schöffski et al., 2022; De Sutter et al., 2023).

We could not find a correlation between the establishment of a model and the type of mutation (primary *KIT* versus secondary *KIT* versus *PDGFRA* versus other mutations) of the tumor sample. In previous years, we tried to expand the platform by collecting several *PDGFRA*-mutated tumor samples and tumor samples of the historically called ‘wild-type’ genotype, i.e. GIST with mutations other than *KIT* or *PDGFRA*. We were able to establish a PDX model with the rather frequent *PDGFRA* exon 18 p.D842V mutation, which is the second model with such mutation reported in the literature to our knowledge (Rossi et al., 2023). Patients with advanced and/or metastatic GIST with this mutation can benefit from avapritinib, but with risk of harmful side effects (Casali et al., 2022). No non-*KIT*/*PDGFRA* GIST PDX model could be established in our platform.

Additionally, although it is described that GISTs with pure epithelioid cell morphology have, in general, worse outcomes, we could statistically not show a better engraftment rate for tumors with this morphology (Joensuu, 2008). It should be noted that no difference was seen in establishment rate between a tumor sample retrieved from a biopsy compared to tumor samples from surgery, meaning that even small tumor pieces can be sufficient for engraftment in mice. We also did not see a difference in the establishment rate of a model comparing systemically pretreated and non-pretreated donors. Different experiences have been described in the literature, with higher failure in tumor engraftment after chemotherapy in lung squamous cell carcinoma to more efficient engraftment of triple-negative breast tumors after systemic therapy (Byrne et al., 2017; Jung et al., 2020). It is plausible to think that the systemic therapy might have decreased the number of viable tumor cells in the tumors (Jung et al., 2020); however, in GIST it is known that systemically pretreated, progressive disease is usually very aggressive, with median progression-free survival rates shorter than 4 weeks (Blay et al., 2020; Serrano et al., 2023). It is likely that more aggressive tumor subclones overcome the treatment, mostly owing to secondary, resistant *KIT* mutations, and these remaining cells after TKI treatment might be sufficient for further tumor growth in the laboratory mice (Liegl et al., 2008). In the models from pretreated donors, we indeed mostly observed secondary mutations (UZLX-GIST9, UZLX-GIST47, UZLX-GIST73, UZLX-GIST76), as well as a primary *KIT* exon 9 mutation (UZLX-GIST2B). GISTs with *KIT* exon 9 mutations are known to be imatinib dose dependent (Debiec-Rychter et al., 2006). Those more resistant tumor cells are probably able to escape treatment and are therefore strong enough to survive in the novel environment of the mouse. However, in those established models from pretreated patients where the GIST PDX model only harbors imatinib-sensitive primary *KIT* mutations (such as UZLX-GIST41), the resistant subclones that required selective TKI pressure in the patient to overgrow sensitive subclones have probably, in turn, been overgrown by TKI-sensitive subclones after absence of this TKI pressure in our mice (Kang et al., 2013; Grunewald et al., 2014).

Twelve tumor samples eventually led to an established PDX model, meaning that after engraftment of the tumor sample (passage 3 and higher), stable histologic and molecular features for at least two passages were observed, similar to those of the original patient's tumor. These findings resulted in a tumor establishment rate of 13.5%. This is in line with the establishment rate of 16.8% for GIST PDX models reported by Na et al. (2020), in which the more immunodeficient non-obese diabetic (NOD) severe combined immunodeficiency disease (SCID) gamma (NSG) mice were used for the first implantation of patient tumor material. Subsequently, Na and colleagues used BALB/c nude mice for successive transplantations, a mouse strain also known for higher engraftment rates than other nude mice (Liu et al., 2023). The reason that we chose NMRI *nu*/*nu* mice is the lower complexity in housing, caretaking and handling of the mice compared to e.g. NSG mice. NMRI *nu*/*nu* mice are only partially immunodeficient and can be housed in a standard barrier facility, in individually ventilated cages. Compared to the establishment rate of other soft tissue sarcoma subtypes in our group, for which we used the same conditions and mice, the establishment rate of our GIST tumor samples is also close to our earlier described 17% (Cornillie et al., 2019). Whereas we developed heterotopic GIST PDX models, Sicklick et al. (2014) generated orthotopic models in order to better study the biology in GISTs. These models potentially recapitulate more accurately the intra-abdominal microenvironment in which clinical GISTs arise, but are more complicated to work with owing to the need for imaging modalities to follow up tumor growth. In our PDX models, the tumors are transplanted subcutaneously; therefore, their growth can be assessed more easily by eye or with calipers, and we also use small-animal imaging for assessment of the lesions if needed. The success rate of xenografting reported by Sicklick et al. (2014) was 84%, which meant that they saw tumor development after transplanting the tumor samples in 84% of cases, independent of the number of passages. They transplanted samples of three PDXs into 14 mice in total, of which 11 mice showed tumor growth in the first passage (passage 0). Five tumor samples in total could be passaged twice, coming from all three PDXs, meaning that all PDXs were successfully passaged up to two times. It is difficult to compare establishment rates with our findings, because the success rates are differently defined and, additionally, the used techniques were very different: the location of implantation (heterotopic), the type of mice (NOD-SCID and NSG mice), and also the number of mice used for initial transplantation of the tumor sample received from the patient (Sicklick et al., 2014). We only transplanted a received patient tumor sample into a maximum of three mice, which gives an acceptable result, keeping in mind the cost and ethical considerations.

To our knowledge, our platform has one of the largest numbers of reported GIST PDX models in the literature, together with Na et al. (2020), which reported 28 established GIST PDX models and Jiang et al. (2016), which reported four GIST PDX models (Jiang et al., 2016; Na et al., 2020). Na et al. (2020) did not describe the presence of PDX models with *KIT* secondary mutations, of which we and Jiang et al. (2016) have a few models available. The creation of a GIST PDX model by other research groups has also been described in studies testing novel drugs or in broader pan-cancer PDX platforms, yet they often include only single cases, with the focus on PDXs of SDH-deficient GISTs and of one *PDGFRA*-mutated GIST (Chijiwa et al., 2015; Powers et al., 2018; Flavahan et al., 2019; Yebra et al., 2022; Rossi et al., 2023). Our PDX models have already been used in numerous *in vivo* drug testing experiments, of which some provided a preclinical rationale and supportive evidence for a number of clinical trials (Floris et al., 2009; Van Looy et al., 2015; Gebreyohannes et al., 2016, 2019a,b; Pulkka et al., 2019; Wang et al., 2020; Schöffski et al., 2022; De Sutter et al., 2023). With the recent expansion of our GIST PDX models, it is hoped that further supportive evidence for clinical trials can be obtained.

In our xenograft platform, tumor characteristics – such as the morphology of the cells, IHC markers and the mutational status of the established GIST models – were identical to the reported patient donor's characteristics and were preserved throughout passaging. In one model, however, the patient's donor tumor showed a primary *KIT* mutation in exon 11 and a secondary mutation in exon 13 (model UZLX-GIST76). The patient’s tumor progressed after receiving imatinib for 1.5 years. We received a part of this tumor after surgical debulking for the creation of a PDX model, and, after analysis of the received tumor sample and the established mouse model, we noticed that, in this received sample and in this model, there was no exon 13 mutation but a heterozygous exon 17 mutation instead. The *KIT* exon 11 and 17 mutations remained stable in this model after several passages. This example confirms again the heterogeneous subclones with diverse secondary mutations that are present in GISTs and, additionally, that some mutational subclones might be more prone to engraftment than others (Liegl et al., 2008).

In conclusion, we have established a platform currently consisting of 12 well-characterized PDX GIST models, reflecting the heterogeneous mutational background of clinical GISTs that can be used for tumor biology studies and preclinical drug testing. Our models are accompanied by full clinical information and with stored archival *ex*-mouse tumor material [cryopreserved in liquid nitrogen, frozen at −80°C and fixed as formalin-fixed, paraffin-embedded (FFPE) and TMA blocks], available for joint research projects with industrial and academic institutions.

## MATERIALS AND METHODS

### Collection of patient tumor samples and clinical data

A thorough description of the collection of patient soft tissue sarcoma tumor samples was published by Cornillie et al. (2019) and still applies to our current GIST PDX models. Fresh tumor samples were collected during a surgical procedure or a clinically indicated biopsy from consenting patients with GIST treated at the University Hospitals Leuven. The collection of the material and its use for xenografting has been approved by the Medical Ethics Committee, University Hospitals Leuven/KU Leuven (Leuven, Belgium; project number S53483) and was performed according to the ethical guidelines set forth in the Declaration of Helsinki. All donors of tissue samples were registered in a clinical database (LECTOR, project number S51495). The database contains a comprehensive dataset of all relevant patient and tumor information. Among other clinical data, the following specific data were collected: sex, age at diagnosis, age at sample collection, origin of tumor sample (biopsy versus surgery, primary versus local relapse versus metastatic lesion), histological and molecular characteristics, confirmed diagnosis, patient's related medical history before and post engraftment, and current clinical status. The patient data related to this project are not publicly available owing to patient privacy requirements. Other data generated in this project are presented in this paper.

### Establishment of GIST PDX models

The GIST fragments collected during a biopsy or during a surgical procedure were immediately implanted subcutaneously as ‘passage 0’ on both flanks of adult female, partially immunodeficient, athymic Naval Medical Research Institute (NMRI) *nu*/*nu* mice (Janvier Labs, Le Genest-Saint-Isle, France). In the case of remaining donor tumor material, pieces for long-term cryopreservation, as well as snap-frozen pieces for molecular analysis, were preserved. When tumor growth reached an estimated volume of ∼200 mm^3^, evaluated by eye, the xenograft was retransplanted to a next generation of mice (‘passaging’). During each passage, one piece of the tumor was snap frozen in liquid nitrogen and preserved at −80°C for molecular assessment of the tumor, and one part was fixed in 4% buffered formaldehyde for histopathological evaluation of the tumor. In the case of remaining tumor material, macroscopically non-necrotic pieces were also preserved in ready-to-use cryovials filled with 10% dimethyl sulfoxide (Sigma-Aldrich, St Louis, MO, USA) in Dulbecco's modified Eagle medium/nutrient mixture F-12 [Life Technologies (Invitrogen), Carlsbad, CA, USA] for long-term cryopreservation. For each passage, the histological characteristics were evaluated. Gene mutations were analyzed for passages 0, 1 and 2, and then every two to three passages. Models received the name ‘UZLX-GISTxx’ with ‘xx’ being a subsequent, chronological number. If a patient donated more than one sample, the number of the first sample was used, and the letter ‘A’, ‘B’, ‘C’, etc. was added. A xenograft was considered ‘successfully engrafted’ whenever a growing tumor could be transplanted to a next generation of mice (passage 1 or higher) and was considered ‘established’ after observing stable histological and molecular features, similar to those of the original patient's tumor, for at least two passages. A model was called ‘unsuccessful’ or ‘non-established’ when none of these criteria were met, and ‘early stage’ when the model was growing but did not reach passage 2 yet.

The laboratory mice were housed in a standard barrier facility, in individually ventilated cages, with food and water available *ad libitum*. The animal work has been approved by the Ethics Committee for Animal Research, KU Leuven (projects P175-2015 and P196-2020) and was performed according to local guidelines and Belgian/European Union regulations.

### Histopathological and molecular characterization of GIST PDX models

Formalin-fixed tumor specimens were embedded in paraffin, and 4 µm sections were cut for H&E staining and IHC analyses. Cell morphology and positivity for GIST-related markers – such as KIT, DOG-1 and CD34 – were evaluated. The human origin of the *ex*-mouse tumors was confirmed by HLA-A immunostaining. The following primary antibodies were used for IHC: anti-KIT (A450229-2, Agilent, Santa Clara, CA, USA), anti-DOG-1 (clone K9, DOG-1-L-CE, Leica Biosystems, Nußloch, Germany), anti-CD34 (M7165, Agilent) and anti-HLA-A (Ab52922, Abcam, Cambridge, UK). Antigen–antibody complexes were visualized using 3,3′-diaminobenzidine (DAB+; Agilent), incubated for 10 min, and slides were counterstained with Gill III Hematoxylin (VWR, Radnor, PA, USA). Stained tissue sections were analyzed using a BX43 microscope (Olympus, Tokyo, Japan). Representative pictures were captured using an Olympus UC30 digital camera and analyzed with Olympus cellSens Dimension imaging software. Histological evaluation was performed by experienced laboratory members in close consultation with a reference sarcoma pathologist (R.S.) at the Department of Pathology, University Hospitals Leuven.

For molecular characterization, DNA was isolated from snap-frozen *ex*-mouse tumor fragments (−80°C) and analyzed using bi-directional Sanger sequencing, as previously described (Debiec-Rychter et al., 2005). Mutational analysis was performed to confirm that the *KIT* and *PDGFRA* mutations were similar to those in the donor sample. Low-coverage whole-genome sequencing to study the genomic stability between passages was performed as previously described (Cornillie et al., 2019).

For RNA sequencing, RNA was isolated from snap-frozen tumor fragments using an RNeasy Mini Kit (74104, Qiagen, Hilden, Germany) with on-column DNase digestion.

Quality control of raw reads was performed with FastQC v0.11.7 (Babraham Bioinformatics, Cambridge, UK). Adapters were filtered using Trimmomatic v0.39 (USADEL LAB, Aachen, Germany). (Bolger et al., 2014) Splice-aware alignment was carried out using spliced transcripts alignment to a reference (STAR; National Human Genome Research Institute of the National Institutes of Health, Bethesda, MD, USA) against the reference genome using the default parameters. After alignment, Binary Alignment Map files were sorted using Samtools v1.18 (Genome Research Limited, Hinxton, UK) (Li et al., 2009; Dobin et al., 2013). Reads aligned to the human genome were then separated using Bamcmp v2.2 (Cancer Research UK Manchester Institute, Manchester, UK) (Khandelwal et al., 2017). Quantification of reads per gene was conducted using FeatureCounts from the Subread package (Bioinformatics Division of The University of Melbourne, Melbourne, Australia) (Liao et al., 2013). Count-based differential expression analysis was performed using the R-based Bioconductor package DESeq2 (Genome Biology Unit, Heidelberg, Germany) (Love et al., 2014). Reported *P*-values underwent adjustment for multiple testing using the Benjamini–Hochberg procedure to control the false discovery rate.

Clinical mutational analysis on patient samples was performed through targeted next-generation sequencing as previously described (Vanden Bempt et al., 2020).

### TMAs

TMAs were prepared from FFPE tumor specimens that were also used for the model characterization and/or analysis for presence of the specific therapeutic target (Jawhar, 2009). Material for TMA construction was assessed during model characterization, when the tumor slides were also evaluated microscopically on H&E-stained slides to check the quality of tumor material. Tumor slides containing sufficient amounts of tumor tissue were selected, and annotations of the selected regions of interest were made on the selected H&E tumor slide. Digital scans of the block were made with TMA Grand Master software (3DHISTECH, Budapest, Hungary/ Sysmex, Norderstedt, Germany), and the annotation of the region of interest was digitally confirmed, aligning the location of the region of interest with the corresponding physical donor blocks. Two to three cores of 1.0 or 1.5 mm in diameter were automatically punched out from the donor block by the TMA Grand Master machine (3DHISTECH/Sysmex) according to the digital annotations. Next, the cores were then relocated to a recipient paraffin block in a precise alignment to form the TMA tissue block. From this constructed block, 4 μm sections were cut, H&E stained and scanned for quality control purposes, and slides for IHC analysis were prepared to use for screening of potential novel diagnostic markers and drug targets. This procedure for TMA creation was described in detail previously by Lee et al. (2021).

### Statistical analysis

For the analysis described in this paper, we tested for correlations between establishment of the model and sex, prior systemic treatment before sampling, disease status at sampling, disease stage according to anatomy and to AJCC tumor/node/metastasis (TNM) classification at the time of sampling, sample origin, and, in the case of a metastatic lesion, as sample origin synchronous versus metachronous metastasis, procedure from which the tumor sample was obtained, the current clinical status of the patient, histological subtype, mitotic count and mutation of the GIST. GEE was used for this analysis to correct for multiple samples per patient in some cases. The Kaplan–Meier method was used to show the overall survival of donor patients for successfully established versus non-established tumors, compared using the log-rank test. Overall survival was defined as the time between first GIST diagnosis and death from the disease. For this survival analysis, only the establishment of the chronologically first-received sample from patients that donated more than one sample was taken into account. Death from an unrelated cause was considered to be a censored observation in our study design. Missing or unknown information and non-established tumor samples were excluded from the analyses. Prism 8 (GraphPad Software, Boston, MA, USA) and Statistical Package for the Social Sciences [SPSS; International Business Machines Corporation (IBM), Armonk, NY, USA] were used for statistical analysis, with *P*≤0.05 considered as significant.

### *In vivo* experiments on XenoSarc models

All established GIST PDX models are available for *in vivo* drug testing experiments. Models for these experiments are usually selected upon their mutational or TKI sensitivity profiles. Drugs can be administered orally via gavaging, by injection intraperitoneally or through the tail vein. During the *in vivo* experiments, tumors are measured by caliper because of their subcutaneous location; however, small-animal imaging such as magnetic resonance imaging, computed tomography, positron emission tomography, optical imaging (including fluorescence, bioluminescence, fibred confocal fluorescence microscopy and optical coherence tomography) and ultrasound can be performed at the Molecular Small Animal Imaging Center (MoSAIC), KU Leuven core facility as applied and reported in our previous projects (Prenen et al., 2006; Floris et al., 2013).

During the experiments, at any timepoint, blood and derived plasma from the xenograft-bearing mice can be collected, as well as organs for FFPE blocks, or frozen at the end of the experiment for the assessment of potential toxicity of the experimental compounds.

## Supplementary Material

10.1242/dmm.052225_sup1 Supplementary information
