# Supplementary material for: Patient-derived xenograft models of gastrointestinal stromal tumors provide a ready-to-use platform for translational research
Source: Dis Model Mech. 2025 Feb 21;18(2):DMM052225. doi: 10.1242/dmm.052225 (PMC11876840; doi:10.1242/dmm.052225)
Supplement: Supplementary information [file dmm-18-052225-s1.pdf]

Table S1. Clinical characteristics of the donors and their tumors per established gastrointestinal stromal tumor (GIST) patient-derived xenograft model.

Model	Disease status of the patient at moment of sampling	Prior treatment before sampling	Procedure from which tumor sample was obtained	Sample origin	Location of primary GIST	Disease stage of the patient at moment of sampling	Current clinical status of patient at last follow up
**UZLX-GIST1**	metastasized	no	surgery	metastasis	gastric	IV	died of disease
**UZLX-GIST2B**	metastasized	yes	biopsy	metastasis	peritoneal	IV	died of disease
**UZLX-GIST3**	primary	no	surgery	primary tumor	gastric	IIIB	died of disease
**UZLX-GIST4**	metastasized	no	biopsy	primary tumor	gastric	IV	died of disease
**UZLX-GIST9**	metastasized	yes	surgery	metastasis	gastric	IV	died of disease
**UZLX-GIST25**	metastasized	yes	biopsy	metastasis	mesenteric	IV	died of disease
**UZLX-GIST41**	metastasized	yes	biopsy	metastasis	unknown	IV	died of disease
**UZLX-GIST47**	metastasized	yes	surgery	recurrence	small intestinal	IV	alive with disease
**UZLX-GIST70**	metastasized	yes	surgery	metastasis	mesenteric	IV	died of disease
**UZLX-GIST73**	metastasized	yes	biopsy	metastasis	small intestinal	IV	died of disease
**UZLX-GIST76**	metastasized	yes	surgery	metastasis	gastric	IV	no evidence of disease
**UZLX-GIST77**	metastasized	yes	surgery	recurrence	gastric	IV	no evidence of disease

Disease Models & Mechanisms • Supplementary information

Table S2. Histopathological characteristics of the established gastrointestinal stromal tumor (GIST) patient-derived xenograft (PDX) models. These characteristics are identical to the donor tumor's morphology, except for one (UZLX-GIST25), where information on the donor sample is missing.

Model	Morphology	Positive immunohistochemical markers
**UZLX-GIST1**	Spindle cell morphology	KIT; DOG-1; CD34
**UZLX-GIST2B**	Spindle cell morphology	KIT; DOG-1
**UZLX-GIST3**	Epithelioid cell morphology, high mitotic activity	KIT; DOG-1; CD34
**UZLX-GIST4**	Spindle and epithelioid cell morphology	KIT; DOG-1; CD34
**UZLX-GIST9**	Spindle and epithelioid cell morphology	KIT; DOG-1; CD34
**UZLX-GIST25**	Spindle cell morphology; necrotic component	KIT; DOG-1; CD34
**UZLX-GIST41**	Spindle cell morphology	KIT; DOG-1; CD34
**UZLX-GIST47**	Spindle cell morphology	KIT; DOG-1; CD34
**UZLX-GIST70**	Spindle and epithelioid cell morphology	DOG-1; CD34
**UZLX-GIST73**	Spindle cell morphology, high mitotic activity	KIT; DOG-1; CD34
**UZLX-GIST76**	Spindle cell morphology, high mitotic activity	KIT; DOG-1; CD34
**UZLX-GIST77**	Spindle and some small round cell morphology, myxoid matrix	KIT; DOG-1; CD34

DOG-1: discovered on GIST-1, CD34: cluster of differentiation 34

Disease Models & Mechanisms • Supplementary information

Table S3. Clinical relevance of our gastrointestinal (GIST) patient-derived xenograft (PDX) models. Drug testing in these models already provided a preclinical rationale and supportive evidence for a number of clinical trials. Partially based on our *in vivo* work, avapritinib is now approved in clinic for the treatment of advanced GIST with the specific *PDFGRA* mutation p.D842V. (Gebreyohannes, Wozniak, *et al.*, 2019) Similarly, IDRX-42 is currently being tested in a phase I trial (NCT05489237). (De Sutter *et al.*, 2023)

**Compound tested *in vivo***	**PDX models used for *in vivo* experiments**	**Clinical trial and/or approvement in clinic**
**IDRX-42** (De Sutter *et al.*, 2023)	UZLX-GIST2B, UZLX-GIST9, UZLX-GIST25	Phase I trial (NCT05489237, ongoing)
**Avapritinib, BLU-285** (Gebreyohannes, Wozniak, *et al.*, 2019)	UZLX-GIST2B, UZLX-GIST3, UZLX-GIST9	Approved, (NCT02508532, NCT03465722) (Trullas-Jimeno *et al.*, 2021)
**Cabozantinib** (Gebreyohannes *et al.*, 2016)	UZLX-GIST2B, UZLX-GIST4, UZLX-GIST9	Phase II trial (NCT02216578) (Schöffski *et al.*, 2020)
**Onalespib, AT13387** (Smyth *et al.*, 2012)	UZLX-GIST4	Phase II trial (NCT01294202)
**IPI-493** (Floris, Sciot, *et al.*, 2011)	UZLX-GIST2B, UZLX-GIST4	Phase I trial (NCT00724425)
**Retaspimycin, IPI-504** (Floris, Debiec-Rychter, *et al.*, 2011)	UZLX-GIST4	Phase III trial (NCT00688766)

Disease Models & Mechanisms • Supplementary information
